# Serologic Evidence of Occupational Exposure to Avian Influenza Viruses at the Wildfowl/Poultry/Human Interface

**DOI:** 10.3390/microorganisms9102153

**Published:** 2021-10-15

**Authors:** Maria Alessandra De Marco, Mauro Delogu, Marzia Facchini, Livia Di Trani, Arianna Boni, Claudia Cotti, Giulia Graziosi, Diana Venturini, Denise Regazzi, Valentina Ravaioli, Fausto Marzadori, Matteo Frasnelli, Maria Rita Castrucci, Elisabetta Raffini

**Affiliations:** 1 Institute for Environmental Protection and Research (ISPRA), 40064 Ozzano dell’Emilia (BO), Italy; 2 Wildlife and Exotic Animal Service, Department of Veterinary Medical Sciences, University of Bologna, 40064 Ozzano dell’Emilia (BO), Italy; mauro.delogu@unibo.it (M.D.); claudia.cotti@unibo.it (C.C.); giulia.graziosi2@unibo.it (G.G.); 3 Department of Infectious Diseases, Istituto Superiore di Sanità, 00161 Rome, Italy; marzia.facchini@iss.it; 4 Department of Food Safety, Nutrition, and Veterinary Public Health, Istituto Superiore di Sanità, 00161 Rome, Italy; livia.di.trani@gmail.com (L.D.T.); arianna.boni@iss.it (A.B.); mariarita.castrucci@iss.it (M.R.C.); 5 Animal Health‒Ravenna, Department of Public Health, Azienda Unità Sanitaria Locale della Romagna, 48124 Ravenna, Italy; diana.venturini@auslromagna.it; 6 Hygiene and Public Health‒Ravenna, Department of Public Health, Azienda Unità Sanitaria Locale della Romagna, 48124 Ravenna, Italy; denise.regazzi@auslromagna.it; 7 Istituto Zooprofilattico Sperimentale della Lombardia e dell’Emilia Romagna B. Ubertini (IZSLER), 48022 Lugo (RA), Italy; valentina.ravaioli@izsler.it (V.R.); faustomarzadori@gmail.com (F.M.); matteo.frasnelli@izsler.it (M.F.); elisabetta.raffini@izsler.it (E.R.)

**Keywords:** avian influenza virus, occupational exposure, human serology, bird-exposed workers, human serosurvey, wildlife/poultry/human interface

## Abstract

Ecological interactions between wild aquatic birds and outdoor-housed poultry can enhance spillover events of avian influenza viruses (AIVs) from wild reservoirs to domestic birds, thus increasing the related zoonotic risk to occupationally exposed workers. To assess serological evidence of AIV infection in workers operating in Northern Italy at the wildfowl/poultry interface or directly exposed to wildfowl, serum samples were collected between April 2005 and November 2006 from 57 bird-exposed workers (BEWs) and from 7 unexposed controls (Cs), planning three sample collections from each individual. Concurrently, AIV surveillance of 3587 reared birds identified 4 AIVs belonging to H10N7, H4N6 and H2N2 subtypes while serological analysis by hemagglutination inhibition (HI) assay showed recent infections caused by H1, H2, H4, H6, H10, H11, H12, and H13 subtypes. Human sera were analyzed for specific antibodies against AIVs belonging to antigenic subtypes from H1 to H14 by using HI and virus microneutralization (MN) assays as a screening and a confirmatory test, respectively. Overall, antibodies specific to AIV-H3, AIV-H6, AIV-H8, and AIV-H9 were found in three poultry workers (PWs) and seropositivity to AIV-11, AIV-H13—still detectable in October 2017—in one wildlife professional (WP). Furthermore, seropositivity to AIV-H2, accounting for previous exposure to the “extinct” H2N2 human influenza viruses, was found in both BEWs and Cs groups. These data further emphasize the occupational risk posed by zoonotic AIV strains and show the possible occurrence of long-lived antibody-based immunity following AIV infections in humans.

## 1. Introduction

It has long been known that wild aquatic birds enable the perpetuation of low-pathogenic (LP) avian influenza viruses (AIVs) that, in poultry, can occasionally evolve into highly pathogenic (HP) strains. The influenza A virus (IAV) gene pool circulates in this major natural reservoir from which novel IAVs can emerge to infect other avian and mammalian species, including humans [1]. To date, 16 hemagglutinin (HA) and 9 neuraminidase (NA) subtypes are known to be harbored in waterbirds, while only H5 and H7 LP strains have the ability to mutate into the HP pathotypes [2].

AIVs can sporadically infect humans, causing mild-to-fatal infections and posing a significant risk to public health in fear of novel influenza pandemics [3]. For this reason, avian influenza surveillance programs have been put in place worldwide. In particular, zoonotic spillovers have been documented for H5N1, H5N6, H5N8, H7N3, H7N4, H7N7, H7N9 HP and H7N2, H7N3, H7N7, H7N9, H9N2 LP AIVs [4,5,6,7]. Virological evidence of bird-to-human transmission have also been reported for H6N1, H10N7, H10N8, and, more recently, H10N3 LPAIV [4,6,8].

Serological surveillance studies may provide an additional informative approach to evaluate the occurrence of AIV human infections that sometimes are undetected due to a relatively short virus shedding and possible mild disease. Indeed, serological studies represent a relevant tool to assess—in a wider time frame—the antibody-based immunity following AIV infections in humans. As reviewed by Sikkema et al. [9], serological evidence of human exposure to AIVs has been found worldwide for thirteen HA—from H1 to H13—and nine NA—from N1 to N9—antigenic virus subtypes. Serological and virological evidence of human infections related to H7 AIVs belonging to both LP and HP strains, have been reported in poultry workers from intensively reared poultry farms of Northern Italy [10,11,12,13].

Close contact with infected birds is considered the main risk factor for either LP and HP AIV infections in humans [6,14], and occupational exposure to avian species can increase this zoonotic risk. From an ecological point of view, AIVs are natural components of wetland ecosystems in which they occupy trophic niches represented by susceptible hosts. However, ecosystem interactions underlie possible spillover of AIVs from natural avian reservoirs to domestic birds [15], thus increasing the related zoonotic risk to workers occupationally exposed to poultry [16,17]. On the other hand, workers who frequently come into contact with wild waterbirds may be directly exposed to AIVs circulating in these natural reservoirs [18].

The main aim of this paper was to assess serological evidence of AIV infection in bird-exposed workers operating in an Italian geographic area considered at high risk for the introduction of avian influenza in poultry farms, being characterized by the abundance of wetlands—habitat of AIV reservoir species—as well as by the presence of small-scale poultry farms representing a potential wildfowl/poultry interface. According to a One Health approach, AIV surveillance data from birds reared in the study area were also integrated and analyzed in the present study.

Our data further emphasize the occupational risk posed by zoonotic AIV strains and reveal the possible occurrence of long-lived antibody-based immunity following AIV infections in a wildlife professional enrolled in this study.

## 2. Materials and Methods

### 2.1. Study Area and Bird Farming System

Domestic birds and bird exposed workers (BEWs) were sampled in a 1128 km^2^ lowland area located in the north of the province of Ravenna, which covers 1858 km^2^ in the Emilia Romagna Region (Northern Italy) and where, as of 1 June 2007, the poultry population was about 3675,000 heads housed in 136 commercial farms. According to the bio-security level (BSL), the bird production system can be classified into: high BSL farms, where birds—light breeders, pullets, laying hens, broilers, cockerels, backyard poultry breeders (*Gallus gallus domesticus*), guinea fowls (*Numida meleagris*), turkeys (*Meleagris gallopavo*)—were intensively reared under indoor conditions, and lower BSL small-scale farms if compared to the first one system. The latter—a total number of 17 small-scale farms—included either turkeys, chickens, ducks, geese, guinea fowl, and quails sold in live bird markets by multi-species dealers/growers or birds housed partially or totally outdoors such as: (i) goose (*Anser anser domesticus*), swan goose (*Anser cygnoides*), muscovy duck (*Cairina moschata*), peking duck (*Anas platyrhynchos domesticus*) breeders; (ii) game birds such as mallard ducks (*Anas platyrhynchos*), pheasants (*Phasianus colchicus*), red-legged partridges (*Alectoris rufa*), rock partridges (*Alectoris graeca*), grey partridges (*Perdix perdix*), quails (*Coturnix coturnix*); (iii) ornamental chickens and ducks, peafowl (*Pavo cristatus*), and gray crowned cranes (*Balearica regulorum*). Backyard poultry for self-consumption was also widespread in the Ravenna province (flocks from a few to 250 heads in 1850 rural farms).

The richness of irrigation canals and ditches, populated by wild waterfowl, characterize the study area whose north-eastern border includes the wetlands of Comacchio Valley and the Po River Delta that represent important sanctuaries for AIV reservoir species.

### 2.2. Sample Collection in Birds and Humans

Between July 2005 and June 2006, virological and serological sample collection from live birds was planned during the ongoing AIV surveillance to examine farms considered at risk for AIV spillover from the wild aquatic bird reservoir to captive-reared avian species (Table 1).

According to the ongoing Regional AIV Surveillance Plan, each farm was considered as one epidemiological unit, including one or more locations from which groups of 10 birds per species-specific population were examined on a monthly basis [19]. Cloacal swabs and oropharyngeal swabs were collected from Anseriformes and Galliformes species, respectively. Sera were taken from geese, ducks (peking or muscovy ducks), mallards, domestic duck hybrids (mulard ducks), pheasants, guinea fowls, red-legged partridges, gray partridges, quails, ornamental chickens, and commercial chickens.

Between April 2005 and November 2006, human serum samples were obtained from: (i) BEWs at occupational risk for AIV infection in the study area, including poultry workers (PWs), veterinarians exposed to birds reared partially or totally outdoor, veterinarians and technicians involved in avian influenza diagnostics; (ii) a wildlife professional (WP) handling wild aquatic birds in major Italian wetlands (Emilia Romagna and Tuscany regions); (iii) control subjects (Cs) represented by workers from the Ravenna province, not-exposed to birds through occupational and/or recreational activities. To evaluate possible seroconversions—i.e., participants who were AIV seronegative at study entry and became seropositive at follow up or participants who were AIV seropositive at study entry and had at least a fourfold increase in antibody levels at follow up—three blood sample collections were planned from each individual: the first one between April and August 2005; the second one in December 2005; the third one between May and November 2006. In the case of the WP, who was enrolled in the study during the third sampling period, two subsequent blood collections were performed in July 2008 and April 2017. All individuals who agreed to participate in the study were previously identified through input from animal and human health services, then invited to a meeting organized to explain their role in the study, and finally enrolled on a voluntary basis. Information about socio-demographic and personal data, history of influenza vaccination, clinical signs of influenza-like illness (ILI) and/or conjunctivitis, individual and environmental biological risk factors, such as their occupational history of exposure to birds, was investigated through direct interview, by means of standardized questionnaires.

### 2.3. Animal and Human Rights Statement

According to the Italian law:

(i) no permit and approval were needed for captive-reared birds because the study was conducted as part of the national avian influenza surveillance program;

(ii) no ethical approval was needed for human sample collection because the study was conducted as a part of the occupational health surveillance program. In particular, human blood samples were collected during activities of preventive medicine, routinely carried out on workers, and aimed to implement health surveillance as well as evaluate fitness for work and specific occupational risks. In addition, written informed consent was obtained from all participants in the study in order to protect personal and health data in accordance with the privacy regulations in force at that time.

### 2.4. Experimental Design and Laboratory Tests in Birds

According to the ongoing Regional AIV Surveillance Plan, cloacal and oropharyngeal swabs from birds were examined for influenza A virus isolation immediately after sampling; in addition, sera from Galliformes birds were also immediately tested for H5 and H7 antibody detection by hemagglutination inhibition (HI) assay, according to standard procedures [20]. Residual serum samples and other bird species’ collected sera were stored in aliquots at −20 °C for further analysis.

For influenza A virus isolation, cloacal and oropharyngeal swabs were examined as pools of five to ten specimens, grouped together according to subsequent criteria of homogeneity for: (i) farm; (ii) bird group; and (iii) bird species. Swab pools were processed and then inoculated into 9 to 11 days-old specific pathogen-free (SPF) embryonated chicken eggs, according to standard procedures [20]. After 3 to 4 days of incubation, influenza isolates were identified by both the hemagglutination (HA) test [21] and a double-antibody sandwich enzyme-linked immunosorbent assay (ELISA), specific for the detection of influenza A virus nucleoprotein (virological ELISA) [22]. Allantoic fluids tested positive by both HA and virological ELISA assays were sent to the Italian National Reference Laboratory for Avian Influenza and Newcastle Disease (Legnaro, PD) for antigenic subtype and pathotype characterization.

Stored sera were subsequently screened by a double-antibody sandwich blocking ELISA for antibodies against influenza A virus nucleoprotein (serological ELISA) [23], and samples tested positive were further examined by HI assay for antibodies against fourteen—from H1 to H14—AIV antigenic subtypes using as antigens the viruses circulating in Italy (from the study area, when available, or from other Italian provinces); otherwise AIV reference strains were used (Tables S1 and S2).

The HI assay was performed according to standard procedures [20] by using inactivated viruses, chicken red blood cells (RBCs), and a standardized quantity of HA antigen (4 HA units). Sera were adsorbed with packed chicken erythrocytes before testing in the HI assay to reduce false-negative reactions [21]. Panels of AIVs and antisera used for serological HI test in birds are shown in Table S1. In ELISA and HI serological tests, antibody titers ≥1:8 and ≥1:16 were, respectively, considered positive [20,23].

### 2.5. Experimental Design and Laboratory Tests in Humans

Human sera were screened for antibodies against AIVs from H1 to H14 subtypes using a modified HI assay based on equine red blood cells (ERBC), shown to be more sensitive in detecting human antibodies against AIVs when compared to a classic HI test, based, e.g., on turkey red blood cells (TRBC). HI titers ≥1:10 were considered potentially positive for AIVs [10,11,24,25,26].

Viruses circulating in the farms under study or other Italian strains were preferentially used as AIV antigens; otherwise, AIV reference strains were utilized (Table S3). However, AIVs of H2N2, H5N2, and H9N2 antigenic subtypes were excluded from this panel of antigens, as preliminarily found to be associated with false-positive HI results possibly due to a steric hindrance mediated by antibodies against the N2 subtype neuraminidase [27] of human origin.

Human sera were also tested by a TRBC-based HI assay aimed to detect antibodies against H1N1 and H3N2 seasonal influenza viruses circulating in the human population between 1995 and 2006 (Table S3). The TRBC-based HI assay was performed according to standard laboratory procedures [21] and only individuals showing no HI reactivity (i.e., titers < 1:10) to human seasonal strains were considered for potential seroreactivity against H1 and H3 AIV antigens. For the HI assays, all human sera were previously treated with receptor destroying enzyme (RDE) to remove nonspecific inhibitors and then adsorbed to packed ERBC or TRBC against false-negative results [21].

Human serum samples tested positive by the ERBC-based HI assays were finally tested for confirmation by a virus microneutralization (MN) assay, using panels of selected avian antigens (Table S3). The MN assays were performed according to the WHO protocol [28]. In brief, the serum samples were first heat-inactivated at 56 °C for 30 min, and then 2-fold serial dilutions were made starting at an initial 1:10 dilution in 96 well plates. Fifty microliters of virus inoculum containing 100 TCID_50_ (tissue culture infectious dose) was then added to the wells and incubated at 37 °C with 5% CO_2_ for one hour. After the incubation, MDCK cells were added to each well (100 µL/well) and incubated at 37 °C overnight. The presence of viral protein was detected by an ELISA using an anti-Influenza A Virus nucleoprotein monoclonal antibody. MN titers were defined as reciprocal of the highest dilution of serum that yielded at least 50% neutralization and were calculated according to the formula used by Rowe et al. [29]. According to previous studies on AIV occupational exposure [30,31,32,33]—also taking into account the variable time intervals from the AIV exposure and serum collection (Table 2)—a low threshold of antibody titer (≥1:10) was chosen for both HI and MN serological assays. HI positive sera confirmed by MN assays were considered to be positive for specific antibodies elicited by previous infection with AIVs.

### 2.6. Statistical Methods

The Fisher’s Exact Test (EPISTAT 3.3, Epistat Services, Richardson, TX, USA) was used to test, within each sampling period, differences in the proportion of BEWs and Cs found to be HI-seropositive (HI titers ≥1:10) to the AIVs under study. For each sampling period, geometric mean HI titers (GMTs) were also calculated for each AIV strain used in the ERBC-based HI assay and were compared by exposure type using the two-tailed unpaired Wilcoxon Sum Rank Test (R software version 3.5.2, R Foundation for Statistical Computing, Vienna, Austria). For both tests, the significance threshold was set at a *p*-value < 0.05.

Because of the potential cross-reactivity between H2 subtype hemagglutinins of avian and human origin—the latter related to the Asian H2N2 human influenza virus that disappeared from the human population in 1968 [6]—a frequency age class-based categorization (20–29, 30–39, 40–49, 50–59, and ≥60 years) of GMTs of sera collection from both BEWs and Cs was planned to evaluate seroreactivity to the H2N3 avian antigen. 

## 3. Results

### 3.1. Bird and Human Study Populations

All of the 17 small-scale commercial farms located in the study area were monitored, from July 2005 to June 2006, during the Regional AIV Surveillance Plan (Table 2). As shown in Table 1, the following farm/production categories were included: free-range waterfowl (*n* = 7 farms), land-based game birds (*n* = 6 farms), dealers (*n* = 2 farms), growers (*n* = 2 farms). Each of these farms consisted of one or more locations sharing epidemiological relationships such as common staff, facilities, and management practices.

A total of 2542 cloacal swabs and 1045 oropharyngeal swabs were collected from Anseriformes and Galliformes species, respectively. Overall, 2688 avian sera were taken from 739 geese, 333 ducks (peking and muscovy ducks), 390 mallards, 10 mulard ducks, 755 pheasants, 250 guinea fowls, 21 red-legged partridges, 21 gray partridges, 8 quails, 151 ornamental chickens, and 10 commercial chickens.

Between April 2005 and November 2006, serum samples were collected from 57 BEWs. This study population included: 46 PWs; 4 veterinarians exposed to birds reared totally or partially under outdoor conditions; 3 veterinarians, and 3 technicians involved in avian influenza diagnostics; 1 WP exposed to wild waterfowl for at least 15 years during capture operations in Italian wetlands. Serum samples were also taken from 7 control subjects (Cs) employed in public companies being present in the same study area, and non-exposed to birds through hobbies and recreational activities. At least one of three scheduled blood samples was taken from individuals under study. Data related to the PWs’ occupational exposure to AIVs are shown in Table 2.

Sociodemographic and personal data are shown in Table 3, in which the study subjects at enrollment were categorized according to the age group, gender, nationality, and years of occupational exposure. The mean age of the 57 BEWs was 46.6 years (range 26–76 years), and 66.7% were male. The mean age of the Cs was 38.4 (range 21–52), and 57.1% were male. During the questionnaire administration, very limited information was provided by the study subjects on the history of influenza vaccination and clinical signs of ILI and/or conjunctivitis.

**Figure 1:**
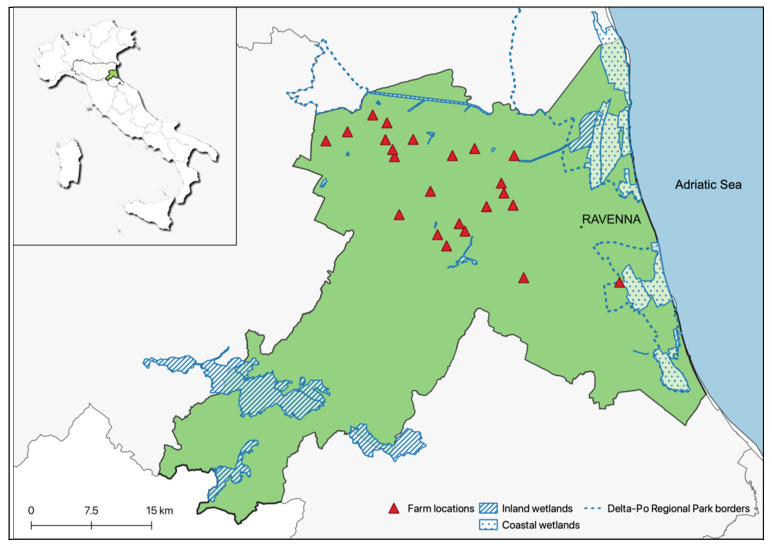
Northern Italy area at high risk for AIV introduction in bird farms, where poultry workers from 23 farm locations were examined by serological assays to assess the occupational zoonotic exposure. See Table 1 and Table 2 for farm details.

As shown in Table 2 and Figure 1, PWs examined operated in 14 out of the 17 farms under study for AIV surveillance in birds (overall 23 locations) and represented 47.9% of the total of occupationally exposed individuals PWs (46/96) employed in small-scale farms. The time worked by each PW in poultry production ranged, in the farm under study, from 1 to 48 years.

### 3.2. Virological and Serological Results from Birds

#### 3.2.1. Avian Influenza Virus Detection, Isolation, and Characterization

Overall, 2542 cloacal swabs and 1045 oropharyngeal swabs were collected from Anseriformes and Galliformes species, respectively. Four of a total of 415 swab pools—processed for virus isolation by inoculation in embryonated chicken eggs—tested Influenza A virus positive by both hemagglutination and virological ELISA assays; the obtained allantoic fluids were further characterized at the National Reference Laboratory for Avian Influenza and Newcastle Disease (Legnaro, Padua, Italy). All the isolates were LPAIVs, belonging to the H10N7, H4N6, and H2N2 AIV antigenic subtypes, found in ducks from three free-range waterfowl farms (A/Mallard/Italy/166998/05, H10N7; A/Duck/Italy/188414/05, H4N6; A/Mallard/Italy/201649/05, H4N6) and in guinea fowls from a bird dealer (A/Guinea fowl/Italy 52892/06, H2N2) (Table 1 and Table 2).

#### 3.2.2. Serological Analyses

A total of 2688 avian sera were collected; of these, 151 and 180—taken respectively from ornamental chickens and guinea fowls and examined only for H5 and H7 subtypes—tested negative by HI assay. The remaining sera were screened by the ELISA test, showing that 64.7% of birds (1525/2357) were seropositive (antibody titers ≥ 1:8) for IAV. Species-specific seroprevalences for IAV nucleoprotein were: 47.2% (349/739) in geese; 79.9% (266/333) in ducks (peking or muscovy ducks); 90% (351/390) in mallards; 10% (1/10) in mulard ducks; 69.0% (521/755) in pheasants; 24.3% (17/70) in guinea fowls; 23.8% (5/21) in red-legged partridges; 19.0% (4/21) in gray partridges; 12.5% (1/8) in quails; 100% (10/10) in commercial chickens. All sera taken from Galliformes birds, immediately examined for H5 and H7 antibody detection according to the Regional AIV surveillance plan, tested negative.

Results of antibody detection against fourteen—from H1 to H14—AIV antigenic subtypes in sera from birds groups in the 17 small-scale commercial farms showed seropositivity against all the HA antigenic subtypes used in the HI assay (Table 2). Notably, seropositivity against new HA subtypes was found in birds by periodic blood sampling, thus providing indirect evidence for recent infections caused by H1, H2, H4, H6, H10, H11, H12, and H13 AIVs in the farms under study.

### 3.3. Serological Results from Humans

Results of the serological screening performed by the ERBC-based HI assay, carried out by using seventeen AIV antigens belonging to fourteen different antigenic subtypes—from H1 to H14—selected as previously described (see Material and Methods section) are shown in Table 4. In particular, either antibody titer frequency or GMT detected in the BEWs and Cs were grouped by sampling period. As shown in Table 3, not all enrolled workers underwent the three planned blood collections. By analyzing HI antibody reactivity against AIVs under study (Table 4), antibody reactivity was observed in both BEWs and Cs sera when tested against each of the H1N1, H2N3, and H3N8 AIV antigens. In contrast, only the BEWs study group included subjects seropositive to the H6N5, H8N4, H9N8, H11N9, and H13N6 AIVs used as antigens in the HI screening test. However, no significant differences in seroprevalence and GMT values were found between BEWs and Cs groups.

During the 1st, 2nd, and 3rd sampling period 37% (20/54), 23.9% (11/46), 11.8% (4/34) of BEWs and 50% (3/6), 33.3% (2/6), 16.7% (1/6) of Cs were seropositive to the A/Mallard/Italy/92151/05 H1N1 strain, respectively; when tested against the A/Mallard/Italy/241431/04 H3N8 strain 55.6% (30/54), 54.3% (25/46), 52.9% (18/34) of BEWs and 66.7% (4/6), 66.7% (4/6), 66.7% (4/6) of Cs were, respectively, found to be seropositive. However, among AIV-H1 and AIV-H3 positive sera, according to the study design, only samples showing no reactivity against homologous H1 and H3 human influenza viruses, listed in Table S3, were further tested by MN assay for specific reactivity against H1 and H3 avian antigens. Overall, we found that eleven BEWs showed antibodies against the H1N1/05 avian strain only (HI titers between 1:10 and 1:80), whereas two BEWs and one subject from the Cs group showed only antibodies to the H3N8/04 avian strain (HI titers between 1:10 and 1:20). Following the MN assay, no seroreactivity to the H1N1/05 strain was confirmed, whereas only 1 of 2 BEWs showed antibodies neutralizing the H3N8/04 AIV (Table 5).

**Figure 2:**
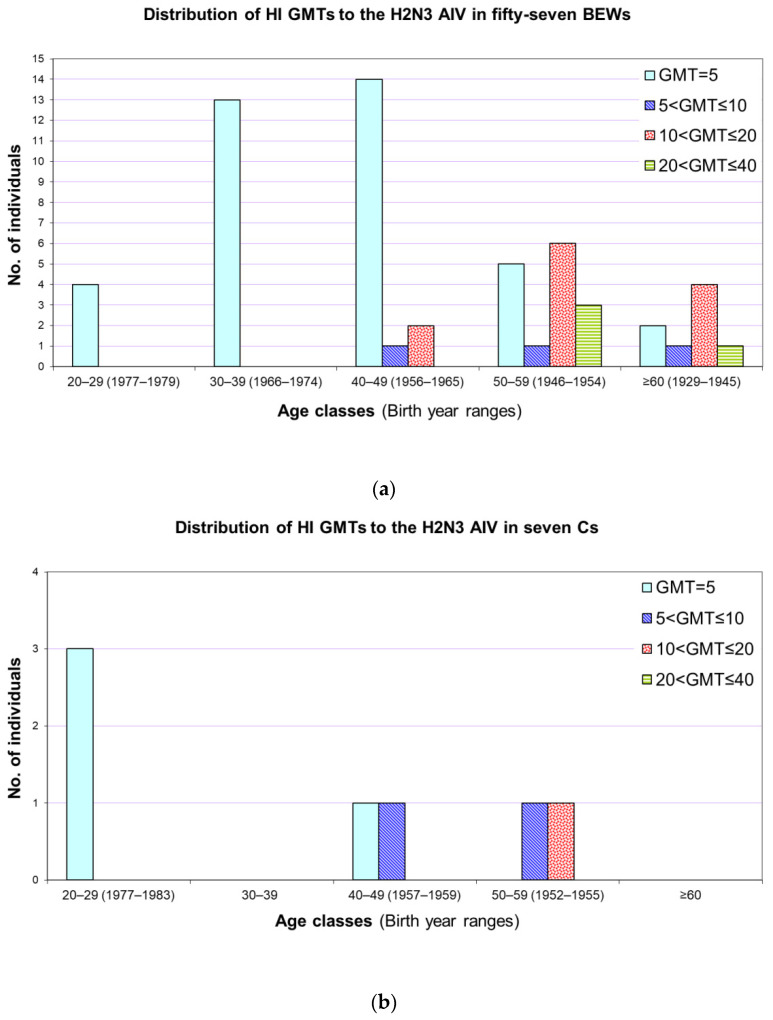
Age class distribution of hemagglutination inhibition geometric mean titers (GMTs) detected against the A/Duck/Germany/1215/73 (H2N3) strain in both (**a**) bird exposed worker (BEWs) and (**b**) control groups (Cs) account for previous exposures to the “extinct” H2N2 human influenza virus.

With regard to the ERBC-based HI assay performed using the H2N3/73 antigen, during the 1st, 2nd, and 3rd sampling period 29.6% (16/54), 34.8% (16/46), 32.4% (11/34) of BEWs and 16.7% (1/6), 50% (3/6), 16.7% (1/6) of Cs were AIV seropositive, respectively. Overall, we found that 19 BEWs and 3 Cs tested seropositive (HI titers between 1:10 and 1:40) at least for one serum sample. The age-class distribution of the GMTs, calculated for BEWs and Cs tested against the A/Duck/Germany/1215/73 (H2N3) strain, is shown in Figure 2. All these H2-seropositivities were associated with the older age classes, whereas individuals belonging to the 20–29 and 30–39 age classes showed H2 GMT < 10, and all of the 51 serum samples from these 22 AIV-H2 positive individuals neutralized the H2N3 strain when tested by MN assay.

Finally, taking into account human sera tested against AIV antigenic subtypes from H4 to H14 (Table 4), we found three BEWs at enrolment showing HI-seroreactivity possibly elicited by AIV-infections. In particular, two PWs showed HI-antibodies to H6N5/72 AIV (with HI titers ranging from 1:10 to 1:40) and to both H8N4/68 and H9N8/03 AIVs (with HI titers ranging from 1:10 to 1:20 and from 1:20 to 1:40, respectively). In addition, one WP was HI-seropositive to both H11N9/05 and H13N6/77 AIVs (with a HI-titer of 1:40 and 1:20, respectively).

Both HI and MN available titers are reported in Table 5. All HI positive titers, except the two lowest ones against the H9N8/03 strain, were confirmed by the MN assay performed with a panel of eight AIV antigens. The antibodies neutralizing AIV-H11 and AIV-H13 strains—detected from November 2006 to July 2008—persisted in the WP for more than a decade until the last blood sampling made in April 2017.

## 4. Discussion

Ravenna province is among Italy’s areas that are at high risk for the introduction of AIVs in poultry being characterized by the abundance of wetlands that are the natural habitat of AIV reservoir species [34], as well as by the presence of small-scale farms representing a potential wildfowl/poultry interface. In this context, the interaction between outdoor housed poultry and wild birds can favor the emergence in reared birds of AIVs harbored in wild avian reservoirs, thus increasing the zoonotic risk to occupationally exposed workers. In this study, BEWs from 14 of 17 small-scale commercial farms were examined for serological evidence of infection by a broad range of AIVs subtypes (from H1 to H14). Moreover, according to a One Health approach, AIV surveillance data from birds reared in all small-scale farms located in the same geographical area were also included to better evaluate the occupational risk posed by zoonotic AIVs.

AIV surveillance activities carried out in reared bird populations between July 2005 and June 2006 showed that only 1% of 415 pools obtained from 3587 cloacal and oropharyngeal swabs were AIV positive. In particular, the influenza virus isolates belonged to the H10N7, H4N6, and H2N2 AIV antigenic subtypes and were found in ducks from the free-range waterfowl farms and in guinea fowls from a bird dealer. However, AIV detection by virus isolation in embryonated chicken eggs may have underestimated the positivity rates of AIVs circulating in the study area when compared with real-time RT-PCR assay [35] later adopted for avian influenza virological surveillance. Indeed, concomitant serological surveillance data showed that 63.5% of birds (1504/2367) were ELISA-positive for influenza A virus antibodies. Results obtained by the serological ELISA screening are consistent with those obtained by the serological HI assay that has overall shown in the examined bird groups seropositivity against all the HA antigenic subtypes (from H1 to H14) used in the assays. In particular, the highest heterogeneity of AIV subtype seropositivity can be observed in free-range waterfowl, whose wild ancestors represent the AIV natural reservoir (farms 1, 3, 5, 6, 15, 16), followed by the dealer and grower production categories, characterized by a multi-host farm system (farms 8, 13) and finally by the land-based game bird farms, housing wild Galliformes species (farms 2, 4, 11, 12, 17) (Table 1 and Table 2). Among various AIV subtypes, seropositivity against H5 and H7 was also observed in Anseriformes birds and likely associated with the previous circulation in wild birds in Italian wetlands [35,36]. In addition, the occurrence of seropositivity against new HA subtypes provided indirect evidence of H1, H2, H4, H6, H10, H11, H12, and H13 AIV circulation in the small-scale farms under study, thus suggesting a realistic risk of AIV infection in the BEWs enrolled.

Our serological data obtained from humans by HI screening assay (Table 4) showed antibody reactivity against each of the H1N1, H2N3, and H3N8 AIV antigens in both BEWs and Cs groups, whereas only the BEWs study group included subjects seropositive to the H6N5, H8N4, H9N8, H11N9, and H13N6 AIVs.

With regard to the H2N3/73 reference strain, the HI seropositivity observed in 19 BEWs and in 3 Cs, and then confirmed by the MN assay, was likely due to cross-reactivity with the Asian H2N2 human influenza virus that disappeared from the human population in 1968 [6]. Indeed, all of these individuals belong to the older age classes, whereas all individuals born after 1968 were seronegative (Figure 2). These results are in agreement with previous reports showing that circulating H2 AIVs are antigenically related to the oldest human H2 viruses [37,38] and may pose a risk of zoonotic infection in young people.

To discriminate AIV-H1 and AIV-H3 positive results possibly related to previous infections with homologous H1 and H3 subtypes of human origin, human serum samples were also tested by HI assays against H1N1 (n = 2) and H3N2 (n = 4) seasonal influenza viruses circulating in the human population between 1995 and 2006. Although 11 BEWs showed seropositivity against the AIV H1N1/05 only and 2 BEWs and 1 subject from the Cs group were seropositive to the AIV H3N8/04 strain only, no seroreactivity to the H1N1/05 stain was confirmed by the MN assay whereas 1 of 2 BEWs showed antibodies neutralizing the H3N8/04 AIV (Table 5).

Serological evidence of human exposure to H3-AIVs has been sporadically reported in Europe and Asia [9] as well as experimentally demonstrated [39]. In the present study, the AIV-H3 seropositivity detected at the 1st and 2nd sampling period in a PW belonging to the “growers” husbandry area (male from the >60 years age group, occupationally exposed to poultry for 2 years) suggests a possible previous exposure to the H3 subtype viruses that were isolated in the study area in December 2003 and October 2004 (Table S2). The second grower PW, sharing the same socio-demographical data with the first one but exposed to birds for 30 years, tested seropositive for the H6N5/72 reference strain at the 1st and 3rd sample collection. In addition, in this case, we observed a trend of antibody titers consistent with previous, non-recent infection. Notably, AIV surveillance data from 4 out of 7 free-range waterfowl farms provided serological evidence of bird exposure to the AIV-H6 subtype, first reported to infect humans in 2013 [40], and since 2002, repeatedly associated with serological evidence of occupational exposure to birds [30,31,32,41,42,43,44,45,46].

The third PW found to be occupationally exposed to AIVs was a 60-year-old female employed for about twenty years in a land-based game birds farm. In particular, she tested HI seropositive to both the H9N8/03 Italian strain and H8N4/68 reference strain in each of the three planned serum collections. The HI seropositivity to the H8 strain, rarely described in humans before [9,32], was confirmed by MN assay performed with the homologous H8N4/06 Italian strain. With regard to the HI antibodies detected with the AIV-H9 subtype, only the third serum collection showed a low antibody level of H9 neutralizing antibodies. Seroprevalence of antibodies to the AIV-H9 subtype, whose pandemic potential is often underestimated [47], has been repeatedly reported in humans as a consequence of exposure to the H9N2 virus circulating in poultry from Asia [43,44,48,49,50,51,52,53,54,55,56,57,58,59,60,61,62,63,64,65,66]; however, the occupational exposure to H9 strains having a different NA surface antigen was sporadically investigated [67]. Serological evidence of previous AIV-H9 infection among bird-exposed workers was also reported in Africa [41,68], America [32,69], and Europe [46,70].

Finally, the WP, who handled free-living wild aquatic birds for at least fifteen years before his first serum collection, was found to be seropositive to AIVs of H11 and H13 antigenic subtypes. Human antibodies against the AIV-H13 subtype, previously detected in Asia, have been scarcely reported [9]. More frequent are cases of seropositivity to the AIV-H11 subtype that, as experimentally shown, has zoonotic potential to mammalian hosts [71]. Serological evidence of H11 infections have been previously reported in poultry workers [32,33,41] and—interestingly—in humans frequently exposed to wild birds for occupational or recreational purposes [69,72]. The WP—male included in the 40–49 years age group at enrollment—showed AIV-H11 and AIV-H13 seropositivity likely due to previous exposure to AIV subtypes circulating in birds in Italy [73]. Different MN titers were observed when using three different AIV-H11 strains—with the highest antibody values detected to the H11N9/00 strains isolated from a wild mallard—and both the anti-H11 and anti-H13 antibody responses persisted in the WP for more than a decade after the first detection (Table 5). The human antibody response to influenza A virus infection can be long-lived, as shown in individuals exposed in the past to the H2N2 virus [74]. Nevertheless, the longevity of human antibody response to AIV infection is less known and—to the best of our knowledge—AIV-specific antibody titers have been previously reported as far as 5 years after H5N1 zoonotic infections [75].

Our study had some limitations which need additional discussion.

Firstly, serosurvey for AIV subtypes H15 and H16 was not conducted due to the lack of availability of the respective reference viral strains for use in serological tests. Indeed, the H16 subtype is not uncommon in certain wild bird species, and recently, it has been noted that some H16 strains bind to the human-type receptor [76,77]. Conversely, AIV with the H15 subtype is rarely detected, but recent data suggest that the avian HA contains inherent mammalian virulence factors [78,79]. Consequently, zoonotic infections with AIV-H15 and AIV-H16 subtypes may occur, and, therefore, it is necessary to include them in AIV surveillance programs.

Secondly, only 57 BEWs and 7 Cs were enrolled. These persons voluntarily participated in the survey; as a consequence, the results are based on an opportunistic sampling strategy. This small sample size decreases the detection sensitivity of a low expected seroprevalence, with a possible missing detection of previous AIV infections in humans. Moreover, a small sample size increases the variability of the prevalence confidence level, with a larger margin of error in estimating the true prevalence [80]. However, our aim was to assess bird-to-human AIV transmission rather than to assess the seroprevalence in exposed individuals.

Thirdly, we chose a low threshold (≥1:10) used in the MN serological assays. This approach, chosen to take into account the variable time intervals from the AIV exposure [30,31,32,33], could account for positive results due to cross-reactivity. Indeed, other studies report higher cut-off titers—up to 1:80—to be more confident that the antibodies are truly of avian virus origin [29,66]. When considering 1:80 cut-off titers for MN assay in our study (Table 5), the seropositivity to AIV-H3, AIV-H6, AIV-H8, and AIV-H11 was still detected in the four BEWs, whereas became undetectable to AIV-9 and AIV-13. Altogether, our results provide further evidence of a potential risk of AIV infection to BEWs in small-scale farms.

## 5. Conclusions

Serosurveys in occupationally exposed workers represent a useful tool to evaluate the zoonotic risk posed by AIVs and allow quantifying associated human infections that may be asymptomatic [6]. Providing new insights into the human antibody responses to AIVs, our findings highlight the crucial role of integrated occupational medicine and veterinary AIV surveillance aimed to further assess the health risk at the wildfowl/poultry/human interface. In this study, some BEWs have shown seropositivity probably due to AIV infection occurring through aerosols, fomites, or contaminated water [81]. Thus, preventive and control measures against zoonotic influenza viruses, including seasonal influenza vaccination of poultry and swine farm workers, are strongly recommended to counteract human infections with influenza A viruses of animal origin and the possible emergence of pandemic influenza virus strains.

## Figures and Tables

**Table 1:** Small-scale commercial farms characterized by the presence of birds housed totally or partially outdoor (Ravenna Province, Northern Italy, 2005–2006).

Farm ID Number	No. of Locations ^†^	Husbrandry/Commercial Area (Type)	Species Reared	No. of Birds
1	1	F-RW (breeding)	Mallards, geese	2000
2	1	L-BGB (breeding)	Pheasants	1600–12,000
3	3	F-RW (breeding)	Ducks, muscovy ducks, geese, swan geese	6000
4	1	L-BGB (breeding)	Pheasants	850
5	1	F-RW (breeding)	Ducks, muscovy ducks, geese, swan geese	4800
6	1	F-RW (restocking, fattening)	Mallards	1600–3200
7	1	F-RW (fattening)	Ducks, geese	1500
8	1	D (ornamental species dealer)	Ornamental ducks and chickens, pigeons, geese, peacocks, turkeys	1000
9	1	G (growing)	Ducks, chickens	500–1000
10	1	L-BGB (hatching, breeding, restocking)	Pheasants, red-legged partridges, quails, grey partridges	5000–45,000
11	1	L-BGB (restocking)	Pheasants	48,000
12	4	L-BGB (restocking)	Pheasants	10,000–100,000
13	4	G (growing)	Ducks, guinea fowls, geese, chickens, turkeys	>10,000
14	1	D (poultry dealer)	Ducks, guinea fowls, geese, chickens, quails	2000–3000
15	2	F-RW (hatching, breeding, restocking)	Mallards	1000–8000
16	1	F-RW (breeding)	Geese	1600
17	1	L-BGB (breeding)	Pheasants, red-legged partridges, rock partridges, grey partridges	1000

^†^, Each farm consisted of one or more locations sharing epidemiological relationships such as common staff, facilities and management practices. F-RW, free-range waterfowl; L-BGB, land-based game birds; D, dealer; G, grower.

**Table 2:** AIV surveillance results in birds and poultry farm workers’ characteristics (Ravenna Province, Northern Italy, 2005–2006).

Farm ID Number ^†^	No. of PWs Examined/Employed (%)	Yearly Average Occupational Exposure (Min–Max)	AIV Isolation in the Farm	HI Seropositivity in Bird Groups (July 2005–June 2006) ^‡, §^
1	1/1 (100)	23	-	**H1**, **H2**, **H6**, H9, H10
2	1/4 (25)	31	-	**H12**
3	3/20 (15)	11 (8–15)	H4N6	**H4**, **H11**, **H13**
4	1/2 (50)	30	-	H14
5	1/4 (25)	15	-	H1, **H4**, H5, **H6**, H7, H8, **H10**, **H11**, H13, H14
6	0/1	na	H4N6	H2, **H4**, H5
7	0/2	na	-	none *
8	1/2 (50)	15	-	H1, H4, H5, H12, H13, H14
9	0/1	na	-	none ^¶^
10	4/5 (80)	20.5 (2–48)	-	none **
11	11/20 (55)	12.8 (4–31)	-	H4
12	2/8 (25)	14 (8–20)	-	H12
13	11/11 (100)	13.2 (2–40)	-	**H2**, H9, H12
14	1/3 (33.3)	30	H2N2	none ^¶^
15	2/2 (100)	1 (1–1)	H10N7	H1, H2, **H4**, **H6**, H9, **H10**, **H12**, H13
16	1/3 (33.3)	8	-	H1, H2, H3, H4, H5, H6, H7, H11, H12, **H13**
17	6/7 (85.7)	15.3 (4–25)	-	H10, **H12**

^†^, See Table 1 for farm details; AIV, avian influenza virus; PWs, poultry workers; HI, hemagglutination inhibition; ^‡^, see Table S1 for AIV antigens and antisera used in the HI assay; ^§^, groups of 10 birds per species-specific population were virologically and serologically tested on a monthly basis; na, not available; -, no AIV virus isolation; *, blood samplings were performed only two times; **, blood samplings were performed only seven times; ^¶^, sera only tested for H5 and H7 AIVs by HI assay. Seropositivity against new HA subtypes, detected in bird farms by periodic blood sampling, is in bold font.

**Table 3:** Characteristics of study subjects at enrolment.

Variable	1st Sampling Period ^†^		2nd Sampling Period ^‡^		3rd Sampling Period ^§^	
	BEWs, *n* = 54	Cs, *n* = 6	BEWs, *n* = 46	Cs, *n* = 6	BEWs, *n* = 34	Cs, *n* = 6
Age group ^ (%)						
20–29	4 (7.4)	2 (33.3)	4 (8.7)	2 (33.3)	0	3 (50)
30–39	12 (22.2)	0	11 (23.9)	0	8 (23.5)	0
40–49	16 (29.6)	2 (33.3)	12 (26.1)	2 (33.3)	13 (38.2)	2 (33.3)
50–59	14 (25.9)	2 (33.3)	14 (30.4)	2 (33.3)	9 (26.5)	1 (16.7)
≥60	8 (14.8)	0	5 (10.9)	0	4 (11.8)	0
Gender (%)						
Male	35 (64.8)	3 (50)	27 (58.7)	3 (50)	23 (67.6)	4 (66.7)
Female	19 (35.2)	3 (50)	19 (41.3)	3 (50)	11 (32.4)	2 (33.3)
Nationality (%)						
North Africa	3 (5.6)	0	3 (6.5)	0	2 (5.9)	0
West Africa	4 (7.4)	0	3 (6.5)	0	3 (8.8)	0
Italy	47 (87)	6 (100)	40 (87)	6 (100)	29 (85.3)	6 (100)
YR of OE (%)						
≤2	5 (9.3)	na	3 (6.5)	na	0	na
2–10	17 (31.5)	na	15 (32.6)	na	9 (26.5)	na
10–20	20 (37)	na	17 (37)	na	18 (52.9)	na
20–30	8 (14.8)	na	7 (15.2)	na	4 (11.8)	na
>30	4 (7.4)	na	4 (8.7)	na	3 (8.8)	na

BEWs, bird exposed workers; Cs, controls, non-exposed workers; ^†^, sampling period between April and August 2005; ^‡^, sampling period December 2005; ^§^, sampling period between May and July 2006 with the exception of one wildlife professional sampled for the first time in November 2006; ^, Age group assignment was made at the first individual blood collection; YR, year range; OE, occupational exposure: na, not applicable.

**Table 4:** HI antibody reactivity of BEWs and Cs sera against AIV antigenic subtypes from H1 to H14 *.

Titer against	1st Samplig Period **		2nd Sampling Period **		3rd Sampling Period **	
AIV Strain *	BEWs	Cs	BEWs	Cs	BEWs	Cs
H1N1/05						
<1:10	34	3	35	4	30	5
1:10	1	0	1	0	0	0
1:20	11	0	1	2	2	1
1:40	5	2	9	0	1	0
1:80	3	1	0	0	1	0
GMT	9.5	15.9	7.9	7.9	6.3	6.3
H2N3/73						
<1:10	38	5	30	3	23	5
1:10	8	1	9	2	10	1
1:20	7	0	4	1	1	0
1:40	1	0	3	0	0	0
GMT	6.9	5.6	7.4	7.9	6.4	5.6
H3N8/04						
<1:10	24	2	21	2	16	2
1:10	9	2	12	2	6	2
1:20	12	2	8	1	6	2
1:40	7	0	3	1	4	0
1:80	2	0	2	0	2	0
GMT	11.1	10.0	9.9	11.2	10.8	10.0
H4N6/05						
<1:10	54	6	46	6	34	6
GMT	5	5	5	5	5	5
H5N9/97						
<1:10	54	6	46	6	34	6
GMT	5	5	5	5	5	5
H5N3/00						
<1:10	54	6	46	6	34	6
GMT	5	5	5	5	5	5
H5N1/05						
<1:10	54	6	46	6	34	6
GMT	5	5	5	5	5	5
H6N5/72						
<1:10	53	6	46	6	33	6
1:10	0	0	0	0	1	0
1:20	0	0	0	0	0	0
1:40	1	0	0	0	0	0
GMT	5.2	5	5	5	5.1	5
H7N1/99						
<1:10	54	6	46	6	34	6
GMT	5	5	5	5	5	5
H7N3/02						
<1:10	54	6	46	6	34	6
GMT	5	5	5	5	5	5
H8N4/68						
<1:10	53	6	45	6	33	6
1:10	1	0	1	0	0	0
1:20	0	0	0	0	1	0
GMT	5.1	5	5.1	5	5.2	5
H9N8/03						
<1:10	53	6	45	6	33	6
1:10	0	0	0	0	0	0
1:20	1	0	1	0	0	0
1:40	0	0	0	0	1	0
GMT	5.1	5	5.2	5	5.3	5
H10N7/05						
<1:10	54	6	46	6	34	6
GMT	5	5	5	5	5	5
H11N9/05						
<1:10	54	6	46	6	33	6
1:10	0	0	0	0	0	0
1:20	0	0	0	0	0	0
1:40	0	0	0	0	1	0
GMT	5.0	5.0	5.0	5.0	5.3	5.0
H12N5/76						
<1:10	54	6	46	6	34	6
GMT	5	5	5	5	5	5
H13N6/77						
<1:10	54	6	46	6	33	6
1:10	0	0	0	0	0	0
1:20	0	0	0	0	1	0
GMT	5	5	5	5	5.2	5
H14N5/82						
<1:10	54	6	46	6	34	6
GMT	5	5	5	5	5	5

*, See Table S3 for AIV antigens and antisera used in the HI assay; **; See Table 3 for sampling period details; HI, hemagglutination inhibition; AIV, avian influenza virus; BEWs, bird exposed workers; Cs, controls, non exposed workers; GMT, geometric mean HI titer. In GMT calculation, an HI reciprocal titer <10 was assigned a value of 5. HI titers ≥1:10 were considered positive.

**Table 5:** Study subjects at enrollment considered AIV seropositive ^§^.

Sex	Age Group	BEWs Type	BEY	AIVs Used in HI	HI Titer			AIVs Used in MN *	MN Titer				
					Sampling Period				Sampling Period				
					1st **	2nd **	3rd **		1st **	2nd **	3rd **	4th ^^^	5th ^^^^
F	50–59	L-BGB	19	H8N4/68	10	10	20	H8N4/06	93.0	na	204.0	na	na
				H9N8/03	20	20	40	H9N8/03	<10	<10	27.5	na	na
M	>60	G	30	H6N5/72	40	na	10	H6N5/72	430	na	292	na	na
M	>60	G	2	H3N8/04	20	20	na	H3N8/04	181.0	147.0	na	na	na
M	40–49	WP	15	H11N6/56	na	na	80	H11N6/56	na	na	99.0	98.7	36.5
				H11N9/00	na	na	na	H11N9/00	na	na	307.9	289.2	117.8
				H11N9/05	na	na	40	H11N9/05	na	na	47.0	50.0	14.0
				H13N6/77	na	na	20	H13N6/77	na	na	33.3	31.7	15.2

^§^, AIV-H2 results, excluded from this Table, are shown in Figure 2. BEWs, bird exposed workers; BEY, year number of exposure to birds; HI, hemagglutination inhibition assay; MN, microneutralization assay; *, See Table S3 for AIV antigens and antisera used in the HI and MN assays; **; See Table 3 for sampling period details; ^, July 2008; ^^, April 2017; L-BGB, land-based game birds; G, grower; WP, wildlife professional; na, not available. H8N4/68, A/Turkey/Ontario/6118/68; H8N4/06, A/Mallard/Italy/UNIBO-399/06; H9N8/03, A/Duck & Goose/Italy/332487/03; H3N8/04, A/Mallard/Italy/241431/04; H11N6/56, A/Duck/England/56; H11N9/00, A/Mallard/Italy/UNIBO-249–1/00; H11N9/05, A/Mallard/Italy/48524/05; H13N6/77, A/Gull/Maryland/704/77.

## Data Availability

The data presented in this study are available in the article and Supplementary Material.

## Supplementary Materials

The following are available online at https://www.mdpi.com/article/10.3390/microorganisms9102153/s1, Table S1: Avian influenza viruses (AIVs) and antisera used for HI assays in birds reared in small-scale farms (Ravenna Province, Northern Italy, 2005–2006), Table S2: Avian influenza virus (AIV) strains isolated between 1997 and 2006 from birds under AIV surveillance in the study area (Ravenna Province, Northern Italy), Table S3: Avian and human influenza viruses and antisera used for serological screening by HI assay and/or for MN confirmatory test in BEWs and Cs.
