# Supplementary material for: Serologic Evidence of Occupational Exposure to Avian Influenza Viruses at the Wildfowl/Poultry/Human Interface
Source: Microorganisms. 2021 Oct 15;9(10):2153. doi: 10.3390/microorganisms9102153 (PMC8539340; doi:10.3390/microorganisms9102153)
Supplement: Supplementary file 1 [file microorganisms-09-02153-s001.zip › microorganisms-1396332-supplementary.pdf]

Table S1. Avian Influenza Viruses (AIVs) and antisera used for HI assays in birds reared in small-scale farms (Ravenna Province, Northern Italy, 2005- 2006).

Virus name abbreviation	Virus strain	Antiserum (production host)
H1N1/05	A/Mallard/Italy/92151/05, H1N1*	A/Mallard/Italy/625-21, H1N1 (C)
H2N2/06	A/Guinea Fowl/Italy/52892/06, H2N2*	A/Duck/Germany/1215/73, H2N3 (R)
H3N8/04	A/Mallard/Italy/241431/04, H3N8*	A/Passeriformes/Italy/00, H3N8 (C)
H4N6/05	A/Mallard/Italy/201649/05, H4N6*	A/Mallard/Italy/239453/05, H4N6 (C)
H5N2/93	A/Mallard/Italy/80/93, H5N2	A/Mallard/Italy/80/93, H5N2 (C)
H5N9/97	A/Chicken/Italy/9097/97, H5N9*	A/Chicken/Italy/9097/97, H5N9 (C)
H6N5/72	A/Shearwater/Australia/72, H6N5	A/turkey/Italy/1173/88, H6N2 (C)
H7N1/99	A/Turkey/Italy/6423-1/99, H7N1*	A/Turkey/Italy/2676/99, H7N1 (C)
H7N3/01	A/Mallard/Italy/33/01, H7N3	A/Turkey/Italy/7159/02, H7N3 (C)
H8N4/68	A/Turkey/Ontario/6118/68, H8N4	A/Turkey/Ontario/6118/68, H8N4 (R)
H9N2/66	A/Turkey/Wisconsin/66, H9N2	A/chicken/Italy/1227/86, H9N2 (C)
H10N7/05	A/Mallard/Italy/166998/05, H10N7*	A/Duck/Germany/N/49, H10N7 (R)
H11N9/05	A/Mallard/Italy/48524/05, H11N9*	A/Duck/Italy/309972/04, H11N9 (C)
H12N5/76	A/Duck/Alberta/60/76, H12N5	A/Duck/Alberta/60/76, H12N5 (R)
H13N6/77	A/Gull/Maryland/704/77, H13N6	A/Gull/Maryland/704/77, H13N6 (R)
H14N5/82	A/Mallard/Ast/82, H14N5	A/Mallard/Ast/82, H14N5 (R)

HI, hemaglutination inhibition; *AIV isolated in the farms under study; C, chicken; R, rabbit.

Table S2. Avian Influenza Virus (AIV) strains isolated between 1997 and 2006 from birds under AIV surveillance in the study area (Ravenna Province, Northern Italy).

**Virus strain**	**AIV subtype**	**Isolation date**	**Bird Farm/Production categories**
A/Chicken/Italy/9097/97	H5N9 LPAI	December 1997	G
A/Turkey/Italy/5563/99	H7N1 LPAI	02/08/1999	G
A/Turkey/Italy/6423-1/99	H7N1 LPAI	16/09/1999	G
A/Turkey/Italy/6423-2/99	H7N1 LPAI	16/09/1999	G
A/Chicken /Italy/5823-2/99	H7N1 LPAI	04/09/1999	G
A/Chicken /Italy/1427/00	H7N1 **HPAI**	28/02/2000	RF
A/Chicken /Italy/1285/00	H7N1 **HPAI**	28/02/2000	RF
A/Duck/Italy/284085/03	H1N1	01/12/2003	F-RW
A/Duck/Italy/284090/03	H1N1	01/12/2003	F-RW
A/Goose/Italy/296426/03	H1N1	09/12/2003	F-RW
A/Duck/Italy/301777/03	H3N8	12/12/2003	D
A/Duck/Italy/118380/04	H1N1	21/05/2004	D
A/Mallard/Italy/185118/04	H4N6	09/08/2004	F-RW
A/Duck/Italy/249176/04	H4N2	28/10/2004	F-RW
A/Goose/Italy/249187/04	H4N2	29/10/2004	F-RW
A/Mallard/Italy/241431/04	H3N8	21/10/2004	F-RW
A/Goose/Italy/278359/04	H11N9	29/11/2004	F-RW
A/Duck /Italy/48524/05	H11N9	15/03/2005	G
A/Mallard /Italy/92151/05	H1N1	26/04/2005	F-RW
A/Mallard/Italy/166998/05	H10N7	25/07/2005	F-RW
A/Duck /Italy/188414/05	H4N6	26/08/2005	F-RW
A/Mallard/Italy/201649/05	H4N6	12/09/2005	F-RW
A/Guinea fowl/Italy 52892/06	H2N2	10/03/2006	D
A/Mallard/Italy/215590/06	H3N6	25/09/2006	F-RW
A/Mallard/Italy/215603/06	H3N6	25/09/2006	F-RW
A/Guinea fowl/Italy/261861/06	H4N6	10/11/2006	D
A/Guinea fowl/Italy 274975/06	H4N6	20/11/2006	D
A/Guinea fowl/Italy 275040/06	H4N6	20/11/2006	D

LPAI, low-pathogenic avian influenza; **HPAI**, highly pathogenic avian influenza; G, grower; RF, rural farms; F-RW, free-range waterfowl; D, dealer

Table S3. Avian and human influenza viruses and antisera used for serological screening by HI assay and/or for MN confirmatory test in BEWs and Cs

Virus name abbreviation	Virus strain	Antiserum (production host)
Avian		
H1N1/05	A/Mallard/Italy/92151/05, H1N1*	A/Mallard/Italy/625-21/2000, H1N1 (C)
H2N3/73	A/Duck/Germany/1215/73, H2N3	A/Duck/Germany/1215/73, H2N3 (R)
H3N8/04	A/Mallard/Italy/241431/04, H3N8*	A/Passeriformes/Italy/00, H3N8 (C)
H4N6/05	A/Mallard/Italy/201649/05, H4N6*	A/Mallard/Italy/239453/05, H4N6 (C)
H5N9/97	A/Chicken/Italy/9097/97, H5N9*	A/Chicken/Italy/9097/97, H5N9 (C)
H5N3/00	A/Mallard/Italy/208/00, H5N3	A/Duck/Italy/26474/04, H5N3 (C)
H5N1/05	A/Mallard/Italy/228090/05, H5N1	A/Mallard/Italy/228090/05, H5N1 (C)
H6N5/72	A/Shearwater/Australia/72, H6N5	A/turkey/Italy/1173/88, H6N2 (C)
H7N1/99	A/Turkey/Italy/6423-1/99, H7N1*	A/Turkey/Italy/2676/99, H7N1 (C)
H7N3/02	A/Turkey/Italy/214845/02, H7N3	A/Turkey/Italy/7159/02, H7N3 (C)
H8N4/68	A/Turkey/Ontario/6118/68, H8N4	A/Turkey/Ontario/6118/68, H8N4 (R)
H8N4/06	A/Mallard/Italy/UNIBO-399/06, H8N4	A/Turkey/Ontario/6118/68, H8N4 (R)
H9N8/03	A/Duck & Goose/Italy/332487/03, H9N8	A/chicken/Italy/1227/86, H9N2 (C)
H10N7/05	A/Mallard/Italy/166998/05, H10N7*	A/Duck/Germany/N/49, H10N7 (R)
H11N6/56	A/Duck/England/56, H11N6	A/Duck/England/56, H11N6 (R)
H11N9/00	A/Mallard/Italy/UNIBO-249-1/00, H11N9	A/Duck/Italy/309972/04, H11N9 (C)
H11N9/05	A/Mallard/Italy/48524/05, H11N9*	A/Duck/Italy/309972/04, H11N9 (C)
H12N5/76	A/Duck/Alberta/60/76, H12N5	A/Duck/Alberta/60/76, H12N5 (R)
H13N6/77	A/Gull/Maryland/704/77, H13N6	A/Gull/Maryland/704/77, H13N6 (R)
H14N5/82	A/Mallard/Ast/82, H14N5	A/Mallard/Ast/82, H14N5 (R)
Human		
hu-H1N1/99	A/New Caledonia/20/99, H1N1	A/New Caledonia/20/99, H1N1 (C)
hu-H1N1/06	A/Solomon Islands/3/06, H1N1	A/Solomon Islands/3/06, H1N1 (S)
hu-H3N2/95	A/Nanchang/933/95, H3N2	A/Nanchang/933/95, H3N2 (C)
hu-H3N2/99	A/Moscow/10/99, H3N2	A/Moscow/10/99, H3N2 (C)
hu-H3N2/04	A/California/7/04, H3N2	A/California/7/04, H3N2 (C)
hu-H3N2/05	A/Wisconsin/67/05, H3N2	A/Wisconsin/67/05, H3N2 (C)

HI, haemaglutination inhibition assay; MN, microneutralization test; *Avian influenza viruses isolated in the farms under study; BEWs, bird-exposed workers; Cs, controls non-exposed workers; C, chicken; R, rabbit; S, sheep.
